# Proceedings of the Fifth Meeting of the American Medical Association

**Published:** 1852-06

**Authors:** 


### EDITORIAL DEPARTMENT.

Presuming that our readers will be better satisfied with a full account of the proceedings of the National Association, than with contributions from our pen, we yield the entire Editorial, and a portion of the Eclectic department of the present No. for that purpose. We are indebted for the following authentic report to the conrtesy of Dr. Gooch, editor of the Stethoscope, and one of the secretaries of the Am. Med. Association:

We find that our limits oblige us to omit portions of the proceedings. The parts omitted will appear in our n xt number.

*Proceedings of the Fifth Meeting of the American Medical Association.*

TUESDAY, May 4, 1852.

The association met in the Second Presbyterian Church at 11 o'clock—the president, Dr. Moultrie, in the chair.

Dr. James Beale, president of the Medical Society of Virginia, and chairman of its committee of reception, welcomed the delegates to the city of Richmond.

Dr. Haxall, chairman of the committee of arrangements, read a list of the delegates who were present, and who answered to their names as follows:—From Maine 2; N. Hampshire 1; Massachusetts 17; Rhode Island 6; Connecticut 9; New York 28; New Jersey 8; Pennsylvania 33; Delaware 3;

Maryland 10; Virginia 90; North Carolina 5; South Carolina 13; Georgia 4; Alabama 4; Louisiana 2; Tennessee 2; Kentucky 8; Ohio 10; Michigan 1; Illinois 3; Missouri 6; Iowa 1; District of Columbia 6; U. S. Navy 1; Foreign 2—275.

Dr. Hays, of Pa., offered the following resolution:

*Resolved*, That a committee of one from each state, to be selected by its own delegation, be appointed to nominate suitable officers for the association.

The resolution having been adopted, the association took a recess of ten minutes, to allow the delegations to appoint the nominating committee.

At the expiration of the recess, the president announced the nominating committee as follows:

Maine—Isaac Lincoln; New Hampshire—Jeremiah Blake; Massachusetts—Jacob Bigelow; Rhode Island—H. W. Rivers; Connecticut—Charles Hooker; New York—Joseph M. Smith; New Jersey—G. R. Chitwood; Pennsylvania—G. W. Norris; Delaware—H. F. Askew; Maryland—G. S. Gibson; District of Columbia—C. Boyle; Virginia—James Beale; North Carolina—James H. Dickson; South Carolina—H. R. Frost; Georgia—C. B. Nottingham; Alabama—A. Lopez; Kentucky—W. L. Sutton; Missouri—C. A. Pope; Ohio—D. Tildon; Illinois—D. Brainard; Michigan—Z. Pitcher; Iowa—J. H. Ranch; Tennessee—Paul F. Eve.

The president requested the secretary to call the roll.

Dr. Cox, of Md., offered the following resolution:

*Resolved*, That when the roll be called, each member shall *rise* in his place and answer to his name.

The resolution was not adopted.

The secretary then proceeded to call the roll, and the members present having answered to their names, the president delivered a lengthy and able address.

The nominating committee reported the following as officers of the association:

*For President*—Beverley R. Wellford, of Va.

*For Vice-Presidents*—Jonathan Knight, of Conn., James W. Thomson, of Delaware, Thos. Y. Simons, of South Carolina, and Chas. A. Pope, of Miss.

*For Treasurer*—Dr. Francis Condie, Pa.

On motion of Dr. Atlee, of Pa., it was

*Resolved*, That the officers thus nominated be and are hereby elected the officers of the association for the ensuing year, and that the nominating committee be requested to nominate secretaries, and to decide upon the next place of meeting at as early a period as possible, the present secretaries to retain their offices until other nominations are made.

This resolution having been adopted, the gentlemen nominated were declared the officers of the association for the ensuing year; and on motion of Dr. Atlee, of Pa., a committee of three, consisting of Drs. Atlee, of Pa., Haxall, of Va., and Eve, of Tenn., were appointed a committee to announce his election to Dr. Wellford, and conduct him to the chair.

Dr. Wellford having taken the chair, returned his thanks for the honor conferred upon him.

Dr. F. C. Stewart, of N. Y., offered an invitation to the association to make the city of New York the next place of meeting.

On motion of Dr. Boyle, this and all similar invitations were referred to the committee of nominations.

Dr. Hays, of Pa., offered the following resolution:

*Resolved,* That the report of the committee on the constitution be made the special order for to-morrow morning.

It was moved by Dr. Stille, that the resolution be so amended as to make it the special order for Thursday. This amendment was lost, and the question being taken on the original resolution, it was adopted.

Dr. Hays also offered the following resolution:

*Resolved,* That the report of the committee of publication and on prize essays be made the special order for the afternoon session.

Dr. Phelps, of N. Y., moved that when the association adjourn, it will adjourn to meet at 4½ o'clock this afternoon.

This resolution was adopted.

Dr. Haxall, chairman of the committee of arrangements, offered the following preamble and resolution, which were unanimously adopted:

The American medical society in Paris being so constituted that it would be entitled to representation if it existed in this country, and as it is recognized abroad as an American institution—

*Resolved,* That the delegates accredited to the association by the American medical society in Paris be and are hereby invited to take seats in this body.

Dr. Drake read the following resolutions, which were laid on the table; and on motion, the association adjourned till 4½ o'clock, P. M.

1. *Resolved,* That every report on a medical or other scientific subject, shall be referred to a select committee, to be read, analyzed and reported on to the association; said select committee indicating its general character and worthiness of publication, provided the authors of every report shall have the right of appealing to the association.

2. *Resolved,* That no report shall be read before the association until it has been examined and reported on by the committee to which it may be referred; nor then but under an order of the association.

3. *Resolved,* That no report shall be published in the Transactions of the association but in virtue of its order.

4. *Resolved,* That all professional and other scientific communications made to the association, shall be referred and treated like the reports of committees.

5. *Resolved,* That the president, vice presidents and secretaries of the association shall be charged with the appointment of the aforesaid committees, being themselves eligible for such appointments.

6. *Resolved,* That the authors of all reports and papers aforesaid, shall have the privilege of reading and explaining the same before the committees.

---

AFTERNOON SESSION.

Dr. B. R. Wellford called the association to order at 4½ o'clock, P. M.

Dr. D. Paul Lajus offered the following resolution, which was unanimously adopted:

*Resolved,* That Dr. Brown Sequard, of Paris, be invited to occupy a seat among the delegates at the present meetings of the association.

Dr. Paul F. Eve, from the committee on nominations, then reported that the committee had *resolved*—

1. That St. Louis be designated as the place for the meeting of the association in 1853.

2. That Drs. P. C. Gooch, of Virginia, and John S. Moore, of Missouri, be nominated for secretaries.

On motion, the report was laid on the table.

Dr. Gooch offered the following resolution, which was rejected:

*Resolved*, That the members of the press be admitted to seats on the floor, and that a committee of three be appointed to raise by voluntary subscription a sum sufficient to defray the expenses of reporting and publishing the proceedings of the meetings, and to make an arrangement for such.

Dr. Isaac Hays read the report of the committee on publication and the reports of the treasurer.

The reports were received, and the following resolutions, appended to the report of the committee of publication, were put and unanimously adopted:

1. *Resolved*, That the assessment for the present year shall be *three dollars.*

2. *Resolved*, That the committee of publication be authorized to fix the price at which the Transactions for the present year will be furnished to such of the members of the association as shall remit the amount decided upon by the committee, within a specified time, (to be fixed also by them.) And that it shall be the duty of the said committee to issue a circular informing the members of the terms upon which the Transactions will be furnished to them.

3. *Resolved*, That the committee be authorized to take such measures in relation to the disposal of the copies of the Transactions remaining after all such members are supplied as shall comply with the terms set forth in the circular of the committee, as they may deem expedient.

On motion of Dr. Ives, the vice presidents were requested to take seats allotted to them in front of the president's chair.

Dr. Hayward presented the report from the committee on prize essays, and broke the seal of the paquet containing the name of the author of the essay, entitled "*On Variations of Pitch in Percussion and Respiratory Sounds, and their Application to Physical Diagnosis*," and which was deemed worthy of the prize. The author proved to be Dr. Austin Flint, of Buffalo, N. Y.. to whom the prize was awarded, and the report was referred to the committee of publication.

The report of the committee on the Medical Botany of the U. States for 1850–'51, from Dr. A. Clapp, chairman, was presented and referred to the committee of publication.

Dr. Drake called up his resolutions offered at the morning session, which were read and discussed. On motion of Dr. Lopez, of Alabama, they were indefinitely postponed.

The reports from the regular standing committees were then called for in order, and were severally laid over or continued. Letters were read from Dr. J. B. Johnson, of Missouri, asking to be excused from further duty as chairman of the committee on epidemic erysipelas, which was granted; and Dr. Thomas Reyburn, of Missouri, asking that the committee on the epidemics of Missouri, Illinois, Iowa, and Wisconsin, be continued, which was also granted.

Dr. Ro. W. Haxall, of Virginia, read a short report of the progress of the committee on the epidemics of Virginia and North Carolina, and asked to be continued, which request was granted.

Dr. Wm. A. Patterson extended to the association an invitation from W.

P. Tunstall, president of the Richmond and Danville railroad company, to an excursion on their road on Friday, 7th May, which was accepted; and, on motion, the thanks of the association were voted to the company.

Dr. Askew moved that when the association adjourn, it adjourn till 9 o'clock on Wednesday, and that it sit from 9 A. M. till 2 P. M. Carried.

On motion of Dr. Gooch, the editorial corps were invited to take seats on the floor.

On motion, the association then adjourned.

---

WEDNESDAY, May 5, 1852.

The association met at 9 o'clock—the president, Dr. Wellford, in the chair.

The minutes were read and approved.

The secretary informed the association that he had enclosed copies of the preambles and resolutions adopted by the association at their sessions of 1850-51, relative to assimilated rank of the medical staff of the army and navy, to the several departments ordered by the resolution. From Dr. Harris, chief of the bureau of medicine and surgery, he had received a letter approving of the course of the association, which letter was read.

Dr. Pinkney, of the navy, asked leave to read a memorial which he had prepared to present to congress, on the subject of assimilated rank. Leave being granted, the memorial was read and explained by its author.

Dr. Cox, of Maryland, offered the following resolutions:

*Resolved unanimously*, That this association approves the memorial emanating from Surgeon Ninian Pinkney of the United States navy, and respectfully asks of congress a calm and dispassionate consideration of its contents; and we, the representatives of the medical profession in the United States, will anxiously await a decision, confidently believing that the relief asked for in the memorial on behalf of the medical corps of the navy, will be granted.

That it is a matter of great interest to the medical profession at large that an act of congress be formally incorporated into the national legislation, and at the present session, which shall define clearly and definitely the relative rank of the medical officers of the navy.

That the bill proposed by Surgeon Ninian Pinkney is approved by this convention, and earnestly recommended as forming a proper and equitable basis for an adjudication of the relative rank, and that this convention will regard any scale less satisfactory to the medical officers of the navy, as unjust to them, and degrading to the profession at large.

That the secretary of this convention be directed to address a copy of these resolutions, together with the memorial of Dr. Pinkney, to the secretary of the navy and the presiding officers of both houses of congress.

On motion of Dr. Yandell of Kentucky, these resolutions were referred to a committee of three, to be appointed by the president.

Dr. Atkinson, of Virginia, offered the following resolution:

*Resolved*, That we have listened with great pleasure to the able and eloquent remarks of Dr. Ninian Pinkney, in vindication of the honor and interests of the profession, and that we will second his efforts to obtain justice at the hands of congress by every means in our power; which was referred to the same committee.

Dr. Hayward, of Boston, offered the following resolution:

*Resolved*, That no member of the association be allowed to speak longer than ten minutes at a time, nor more than twice on the same subject.

Which was unanimously adopted.

Dr. Simmons, of S. C., offered the following preamble and resolutions:

The accumulation of passengers who are emigrants, crowded in ships coming to our shores from foreign ports, having in a great many instances numerous cases of aggravated fever, many of which prove fatal, and likewise producing similar results at the lazarettoes, and even cities; the number, likewise, of sick arriving from California, and some of the South American ports, and the fact that none of these vessels are required by law to have physicians or surgeons on board, seem deserving of our attention as conservators of health, and as an act of humanity and duty on the part of the American medical association, to bring these facts respectfully to the consideration of congress, and to request its legislation thereon.

*Be it therefore resolved*, That the American medical association do memorialize congress to require all vessels carrying steerage passengers on the sea, to have a surgeon on board.

*Resolved further*, That a committee of this association be appointed to draw up a memorial to congress, making such suggestions as it may deem fit as regards the importance of this measure.

On motion, of Dr. Wood, of Pa., the resolutions were laid on the table for the present.

Dr. Storer asked a suspension of the regular order, to enable him to bring to the notice of the association a scurrilous attack upon him as the chairman of the committee on obstetrics, which he pronounced to be malignant, vindictive and false, and which he would not have noticed had it been directed against him personally.

Dr. J. B. Flint, of Kentucky, proposed the following as an alteration of the constitution, which, according to rule, was laid over till the next meeting:

It is proposed to alter the constitution, in the fifth article of it, so as to provide, that instead of the annual volume of Transactions, the association may establish and maintain a quarterly journal, to be a medium for the publication of its proceedings, and of the most valuable contributions of its members—an organ of resolute and impartial criticism, and an official exponent and advocate of the views of the association on medical science, education and ethics.

The report of the committee on the constitution being the special order, Dr. Hays, chairman of the committee, made a report. Dr. J. H. Yardly, a member of the committee, made a counter report. Much discussion ensued, and many resolutions and amendments were proposed and withdrawn in favor of the following resolution offered by Dr. Thomas, of Maryland, and amended by Dr. Stewart, of New York:

*Resolved*, That the two reports on proposed alterations of the constitution be referred to a committee of three, to be appointed by the chair, with instructions to report to-morrow morning, in definite and proper form, such amendments as will embrace the views set forth in the reports, and such other views as may appear to them advisable.

This resolution was adopted.

Dr. Watson, of New York, offered the following resolutions:

*Resolved*, That the report of the nominating committee, now on the table, be referred back to the said committee, with instructions to report complete on the standing committees, and such other committees as may be requisite for providing business for the association at its next annual meeting.

*Resolved*, That the invitation from the New York delegation for the meeting of the association in the city of New York in May 1853 be accepted, and that the nominating committee be instructed to that effect, and as usual to provide for the appointment of one of the secretaries from among the members residing at the place to be selected for the next annual meeting.

Dr. Stewart, of New York, moved to amend the resolutions, by referring the report of the nominating committee back to the committee without instructions.

This amendment was lost.

After some discussion and the proposal of several amendments, the question was taken on the adoption of the original resolutions, and they were unanimously adopted.

The secretary read the following communication from the New York academy of medicine, which, on motion, was referred to the publication committee and ordered to be printed:

NEW YORK ACADEMY OF MEDICINE,<br>*New York*, April 22d, 1852.

SIR—I have the honor herewith to transmit to you a copy of the preamble and resolutions adopted at a regular meeting of the New York academy of medicine, held April 21st, 1852.

Whereas the cliniques now held at the medical colleges, as at present conducted, are or may be made tributary to the private interests of the professors at the expense of other and younger members of the profession, depriving them, by an odious monopoly, of *practice* and *operations*, and often of *fees*, to which they are justly entitled: Therefore,

*Resolved as the sense of this academy*, That to prescribe or operate upon the legitimate patients of any other physician, knowing them to be such, although done gratuitously at a clinique, is equally unwarrantable and unprofessional, with similar interference with the patients of another in private practice; and in either case, is a violation of the code of medical ethics adopted by this body.

*Resolved*, That the possible perversion of these cliniques to the private emolument of those conducting them, by transferring patients to their private offices, and thus exacting fees from those found able to pay, divests the cliniques of all pretext for professing to be public charities, and should be scrupulously guarded against in all our colleges by stringent rules.

*Resolved*, That a copy of these resolutions be sent to the authorities of the several medical colleges in this city.

The secretary was also instructed to forward a copy of the resolutions to the American Medical Association.

Respectfully yours,

JACKSON BOLTON, M. D.,<br>*Recording Secretary.*

P. CLAIBORNE GOOCH, M. D.,<br>*Sec. Am. Med. Asso., Richmond, Va.*

Dr. Haxall, chairman of the committee of arrangements, offered the following resolution, which was adopted:

*Resolved,* That after to-day the association hold a morning session from 9 o'clock, A. M.. to 3 or 3½ o'clock, P. M., and have no afternoon session.

Dr. Hayward, of Boston, read a letter from Dr. Horatio Adams, of Waltham, Massachusetts, regretting his inability to be present at the meeting, owing to a serious accident, and presenting the report of the committee on the "*action of water on lead pipes, and the diseases resulting from it,*" asking the reference of the report to the committee on publication. The report was accepted and referred.

Drs. Drake, of O., and Rogers, of Va., offered several suggestions in regard to the constitution, which were referred to the committee on that subject.

The chairman of the nominating committee requested that the delegates from states not represented when the committee was organized, should appoint their committee-men forthwith.

Drs. Gwathmey and Watson, of Virginia, Smith, of California, and Beck, of New York, were, on motion, admitted to the floor of the association during its sittings.

Dr. Corbin, of Va., read the following resolution, which he desired to lay on the table for the present:

*Resolved,* That one member from each state represented in this association be appointed a delegate to represent it in the medical associations in Europe, and that they be requested to visit the foreign hospitals, and to report to the next meeting of the association the various improvements in the several branches of science connected with medical education, and in the treatment of diseases in general in foreign countries.

On motion, the association then adjourned.

---

### AFTERNOON SESSION.

The president, Dr. B. R. Wellford, took the chair at half-past 4 o'clock, P. M.

Dr. Drake, of Ky., offered the following:

*Resolved,* That all papers and reports on scientific subjects shall be read to the association before the question of their publication shall be decided.

Dr. Wood, of Pa., opposed the resolution.

Dr. Phelps, of N. Y., offered an amendment, which, together with the resolution, was, on motion of Dr. Thomas of Md., laid on the table.

Dr. Condie, of Pa., presented a paper on chemistry, from a gentleman not a member of the association, and Dr. Drake presented a similar one by Dr. Wright, of Ohio, on the influence upon the health of daguerreotypists of their occupation. On motion of Dr. Condie, they were both referred to a selec committee, consisting of Drs. Ro. E. Rogers, A. T. B. Merritt, and J. R. W. Dunbar, with instructions to report on them to-morrow.

On motion of Dr. G. F. Terrill, of Va., Drs. T. L. Scott and W. H. Fox, o Va., were admitted to seats on the floor.

Dr. Eve, from the committee on nominations, recommended the followng officers for the ensuing year:

*For Secretaries*—Dr. P. Claiborne Gooch, of Va., and Dr. Edward Bead, of N. Y.

*Committee on Publication*—I. Hays, of Pa., P. Cl. Gooch, of Va., E. L Beadle, of N. Y., Isaac Parrish, of Pa., G. Emerson, of Pa., D. F. Condie, of Pa and G. W. Norris, of Pa.

*Committee of Arrangements*—F. Campbell Stewart, John Watson, Wm. Rockwell, James R. Wood, Robert Watts, Jr., Alfred C. Post, John G. Adams and H. D. Bulkley, of New York.

On motion, the report was received, and the gentlemen named were unanimously elected officers of the association for the ensuing year.

The chair then announced the following appointments in compliance with resolutions adopted at the morning session.

*Committee on Amendments to the Constitution*—Dr. F. C. Stewart, of N. Y., Dr. Worthington Hooker, of Conn., and Dr. Robert H. Thomas, of Md.

*Committee on Dr. Cox's Resolutions in regard to the Rank of Medical Officers in the Navy*—Dr. Samuel Jackson, of Pa., Dr. Jonathan Knight, of Ct., and C. C. Cox, of Md.

The report of the committee on "The Blending and Conversion of the Types of Fever" was then read by Dr. A. B. Williman of S. C., (in place of Dr. Dickson, not present.)

On motion, the report was ordered to be printed, and referred to the committee of publication.

Dr. Hayward, of Mass., presented and read the report of the committee on "The permanent Cure of reducible Hernia;" which was ordered to be printed and referred to the committee on publication.

On motion of Dr. Dunbar, of Md., seconded by Dr. Drake, a report of the case of Dr. Jameson, of Baltimore, was requested to be furnished for publication in an appendix to the report.

An application was presented from J. Wells, representative of the interests of the late Dr. Horace Wells, of Hartford, Conn., asking that a committee be appointed to enquire into and to report on the claims of the contestants for the honor of priority in the discovery of the principle of anæsthesia in surgical operations.

The application was laid upon the table.

On motion, the association then adjourned.

---

Thursday, May 6, 1852.

The association was called to order at 9½ o'clock—Dr. Wellford, president, in the chair.

The minutes were read, amended and approved.

On motion of Dr. W. E. Horner, Dr. Beylard, of Paris, was admitted to the floor of the association; and on motion of Dr. Wilson, of Virginia, Dr. W. T. Howard, of N. C., was also admitted.

Dr. Jno. Watson, of N. Y., offered the following resolution:

*Resolved,* That members of the association having questions for scientific enquiry to propose as part of the business for the ensuing year, be requested to submit the same in writing to the chairman of the committee on nominations, and that said committee be requested to report on the nominations of the special scientific committees, with the subjects to be referred to said committees, at its earliest convenience:

Dr. Wood, of Pennsylvania, offered the following amendment:

"And that the nominating committee nominate a committee of five, who shall select special subjects of investigation, and nominate chairmen of the of the committees on these subjects, and also to nominate the members of the committee on voluntary communications." Which was lost.

Dr. Watson's resolution was then adopted.

Dr. Atkinson, of Virginia, moved the following:

*Resolved*, That the thanks of this association are due, and are hereby tendered to Dr. Isaac Hays, for the very efficient and satisfactory manner in which he has discharged the duties of its treasurer, and to Dr. H. W. De Saussure, for the able manner in which he has discharged the laborious duties of secretary.

Dr. Green, of N. Y., offered the following resolutions, which were adopted:

1. *Resolved*, That at all future meetings of this association, all reports of committees, and all contributions on scientific subjects, occupying more than ten pages of quarto post manuscript, be accompanied each by an abstract or synopsis embracing the principle points of such report or paper, which abstract or synopsis may be read before the association.

2. *Resolved*, That the above resolution be transmitted by the secretary to the chairman of each scientific committee.

Dr. Stille, of Pa., moved the following resolutions, which were seconded by Dr. Blatchford, of N. Y., and unanimously adopted:

1. *Resolved*, That the elegant, varied and generous hospitality which the association has enjoyed during its present session, calls for its hearty and unanimous thanks, with the assurance that it can never forget an entertainment, unrivaled even among the festivities of the "Old Dominion."

2. *Resolved*, That the thanks of the association are hereby presented to the Medical Society of Virginia, to the medical profession and citizens of Richmond, to the trustees of the "United Presbyterian Church," to the managers of the Danville railroad, and to the several public institutions of this city, for the hospitable care of these bodies to promote the comfort and amusement of the association.

3. *Resolved*, That the association returns its thanks in an especial manner to the committee of arrangements, for the zeal, intelligence, and good taste displayed in performing its numerous and important duties.

Dr. Simons called up his resolutions in regard to the necessity of surgeons being employed on board of emigrant ships; which were advocated by him and adopted.

Dr. W. Hooker, of Ct., offered the following resolution, which was adopted:

*Resolved*, That special committees on medical education and medical literature be appointed, consisting each of five members, and that the nominating committee be instructed to nominate such committee to this association.

Dr. Sutton, of Ky., moved that a committee of three be appointed, whose duty it shall be to enquire whether any, and if any, what action this association shall take in reference to requesting the congress of the United States to have a large edition of the medical statistics, furnished by the census lately taken, published in a separate form for distribution among the medical profession of the United States, and to report to-day.

The chair announced the committee, to consist of Drs. Simons of S. C., Boyle of D. C., and Sumner of Conn.

On motion of Dr. Condie, of Pennsylvania, it was

*Resolved*, That a committee of five be appointed to examine and report on the communication of Dr. Drake, on the relation between climate and pulmonary consumption.

The committee was announced, to consist of Drs. Condie, R. E. Rogers, J. M. Smith, Moultrie and McGuire.

On motion of Dr. Rockwell, it was

*Resolved*, That the committee appointed to memorialize congress on the subject of compelling passenger vessels to carry surgeons, be directed also to call their attention to the importance of giving to each steerage passenger a certain amount of space between decks.

[We omit the communication from American Medical Society of Paris· shall refer to this society in our next number.]

Dr. Blatchford, of N. Y., offered the following:

*Resolved*, That a committee of three be appointed, to report at the next meeting of the association, on the best means of making pressure in the treatment of reducible hernia, and that Dr. Hayward, of Mass., be the chairman. Carried.

Dr. Usher Parsons, of R. I., offered the following preamble and resolution, which, on motion of Dr. Hays, of Pa., were laid on the table:

Whereas, it is required by law that a chest of medicines shall be furnished to every merchant ship, with suitable directions for their administration; and whereas the pamphlets now in use are written by apothecaries, instead of physicians, and are full of errors: Therefore,

*Resolved*, That a committee of three be appointed to prepare suitabe directions to accompany medicine chests, that shall meet the wants of the officers and seamen in merchant vessels, under the sanction of this association, and report at the next annual meeting.

The report of the committee appointed on yesterday to consider the various propositions which had been made, suggesting amendments to the constitution, being called for, the chairman, Dr. F. Campbell Stewart, of N. Y., read a report and resolutions, which Dr. Hays, of Pa., moved to refer to the committee of publication, with instructions to print.

Drs. J. K. Mitchell, of Pa., and Wm. Hooker, of Conn., discussed the merits of the report, when Dr. Lopez, of Alabama, raised a question of order as to the propriety of a discussion of the merits of the proposition.

The chair decided that the discussion was in order at this stage of the proceeding.

Dr. Lopez, of Ala., appealed from that decision, which appeal was not sustained.

The discussion was then continued at great length by many members.

During the discussion, the following replies were elicited from several gentlemen, by questions propounded by Dr. Watson, of New York:

From Dr. Horner, University of Pa.—The shortest term of medical study in the University required for the doctorate was three years, but that under some few and rare circumstances, a deviation had been permitted as an exception.

Drs. Davis and Rogers, of Virginia University, stated that their laws required no specified time: nine months, and eighteen years of age even, were sufficient, but that two years were generally devoted to the study of medicine by their graduates. They explained the course of instruction at the University at length.

Dr. Huston, of Jefferson Medical College, Philadelphia, said that three full years were required, but that occasions demanded sometimes a departure from the stringent rule.

Dr. Frost, from South Carolina, offered some interesting observations upon the much abused subject of medical education, and insisted that the profession had not retrograded. That there had been a steady and gradual improvement in our medical colleges generally, and brought to the notice of the association the attention which was observed in preparatory education in the medical college of South Carolina, which was highly creditable to the same. His remarks were listened to with attention, and brought forth observations of a like character from other members present.

The report and proposed amendments were received, after having been amended so as to read as follows: [We omit the report.]

### Article I.—*Title of the Association.*

This institution shall be known and distinguished by the name and title of "The American Medical association." It shall be composed of all the members of the medical profession of the United States of good standing, who acknowledge fealty to, and adopt the code of ethics adopted by the association; and its business shall be conducted by their delegates or representatives, who shall be appointed annually in the manner prescribed in this constitution.

Strike out the whole of Article II, referring to "Members," and insert the following:

### Article II.—*Of Delegates.*

§ 1. The delegates to the meeting of the association shall collectively represent and have cognizance of the common interests of the medical profession in every part of the United States, and shall hold their appointment from county, state, and regularly chartered medical societies; from chartered medical colleges, hospitals, and permanent voluntary medical associations in good standing with the profession. Delegates may also be received from the medical staffs of the United States army and navy.

§ 2. Each delegate shall hold his appointment for one year, and until another is appointed to succeed him, and he shall be entitled to participate in all the business affairs of the association.

§ 3. The county, district, chartered and voluntary medical societies shall have the privilege of sending to the association one delegate for every ten of its resident members, and one more for every additional fraction of more than one-half of this number.

§ 4. Every state society shall have the privilege of sending four delegates; and in those states in which county and district societies are not generally organized, in lieu of the privilege of sending four delegates, it shall be entitled to send one delegate for every ten of its regular members, and one more for every additional fraction of more than one-half of this number.

§ 5. No medical society shall have the privilege of representation, which does not require of its members an observance of the code of ethics of this association.

§ 6. The faculty of every chartered medical college acknowledging its fealty to the code of ethics of this association, shall have the privilege of sending one delegate to represent it in the association: *Provided*, That the said faculty shall comprise six professors, and give one course of instruction annually of not less than sixteen weeks, on Anatomy, Materia Medica, Theory and

Practice of Medicine, Theory and Practice of Surgery, Midwifery and Chemistry: *And provided also*, That the said faculty requires of its candidates for graduation—1st. That they shall be twenty-one years of age; 2d. That they shall have studied three entire years, two of which must have been with some respectable practitioner; 3d. That they shall have attended two full courses lectures, (not however to be embraced in the same year,) and one of which must have been in the institution granting the diploma, and also where students are required to continue their attendance on the lectures to the close of the session; and 4th. That they shall show, by examination, that they are qualified to practice medicine.

§ 7. The medical faculty of the University of Virginia shall be entitled to representation in the association, notwithstanding that it has not six professors, and that it does not require three years of study from its pupils, but only so long as the present peculiar system of instruction and examination practiced by the institution shall continue in force.

§ 8. All hospitals, the medical officers of which are in good standing with the profession, and which have accommodation for one hundred patients, shall be entitled to send one delegate to the association.

§ 9. Delegates representing the medical staffs of the United States army and navy shall be appointed by the chiefs of the army and navy medical bureaux. The number of delegates so appointed shall be four from the army medical officers, and an equal number of the navy medical officers.

§ 10. No delegate shall be registered on the books of the association as representing more than one constituency.

§ 11. Every delegate elect, prior to the permanent organization of the annual meeting, and before voting on any question after the meeting has been organized, shall sign the constitution and inscribe his name and address in full, with the title of the institution which he represents.

Dr. Wadsworth, of Pa., offered the following resolution:

*Resolved*, That when the association adjourns, it will be to meet again this afternoon at 4½ o'clock P. M., and that the resolution adopted on yesterday be rescinded so far as it conflicts with this action.

Carried.

Dr. Smith, of N. Y., chairman of committee on nominations, made a report, which was recommitted, on motion of Dr. Patteson, of Va., for correction.

The chair then announced the following committee on Dr. Simons' resolution, in regard to the propriety of memorializing congress to pass some law requiring emigrant vessels to carry surgeons, viz: Dr. T. Y. Simons of S. C., chairman, Pope of Mo., Thompson of Del., Flint of Ky. and Mauran of R. I.

Dr. Knight, of Conn., moved to lay the report on amendments to the constitution on the table, to be taken up and voted on, section by section; which was carried.

The association then adjourned till 4½ o'clock P. M.

---

AFTERNOON SESSION.

Dr. Wellford, president, called the association to order at half past 4 o'clock.

Dr. McIntyre, of N. Y. moved to refer the report on the amendments to the constitution to the publication committee; which was lost.

Dr. Smith, N. Y., chairman of the nominating committee, reported and offered the following resolution, which was received and adopted unanimously.

*Resolved*, that the following gentlemen be appointed:

1. *Committee on Medical Literature*—Rene La Roche, M. D., of Pa., chairman; H. W. De Saussure, M. D., of S. C.; N. S. Davis, M. D. of Ill.; Jacob Bigelow, M. D., of Mass.; Ed. H. Barton, M. D., of La.

2. *Committee on Medical Education*—Zina Pitcher, M. D., of Mich., chairman; Austin Flint, M. D., of N. Y.; J. R. W. Dunbar, M. D., of Md.; James McKeen, M. D., of Maine; D. W. Yandell, M. D., of Ky.

The amendments to the constitution, as embodied in the amended report of the committee at the morning session, were then read, section by section, and after some debate, laid on the table as proposed amendments to the constitution.

During the discussion, Dr. Wilson. of Va., offered the following amendment, which was laid on the table, on motion of Dr. Thomas of Md.:

The faculty of every chartered medical college acknowledging its fealty to the code of ethics, and conforming to the requisitions of this association on the subject of medical education as adopted by this association in 1846, and reiterated at its subsequent meetings, shall have the privilege of sending one delegate to represent it in the association: provided that the medical faculty of the University of Virginia shall be entitled to representation in this association in consequence of its peculiar organization, but only so long as its peculiar system of instruction and examination shall continue in force.

Dr. Wilson gave notice that the above would be called up at the next meeting of the association as an amendment to the constitution.

Dr. Atlee, of Pennsylvania, moved the following, which was adopted:

*Resolved*, That this association still recommends to the medical colleges the propriety of lengthening their terms of instruction.

On motion, the following resolution was called up for consideration, and adopted:

*Resolved*, That the colleges exclusively of dentistry and pharmacy are not recognized by this association as among the bodies authorized to send delegates to its meetings.

On motion of Dr. Gooch, of Virginia, the two reports from the committee appointed last year to suggest alterations of the constitution, together with that of the committee to which they were referred on yesterday, were referred to the committee of publication, with instructions to print. * * *

[We omit report on rank of surgeons in the navy. We shall publish the report hereafter.]

Dr. Simons of S. C., chairman of the committee raised on Dr. Sutton's resolution, adopted on Wednesday, made the following report:

"The committee appointed, on motion of Dr. Sutton, to enquire what action should be taken to get Congress to publish the medical statistics of the census of the United States separately, to be presented to the medical profession under the auspices of the medical association, recommend that this or some other committee be empowered to memorialize Congress on the same."

On motion of Dr. Gooch, of Va., the report was received, and the same committee was instructed to carry out the recommendation in it.

Dr. W. Hooker, of Ct., read the report of the committee on the epidemics of New England, together with the following recommendation from the chairman of the several committees on epidemics, which was adopted:

In behalf of the committees on epidemics who are present at this meeting of the association, we present the following communication.

In view of the statement made in the report just presented, and of those which will be presented to you in some of the other reports on epidemics, the undersigned, members of a part of the committees on this subject appointed by this association, would recommend to the meeting the following resolutions:

*Resolved*, That the committees on epidemics be constituted in relation to the division into districts as they were the last year, and that they be continued in service during a period of five years.

*Resolved*, That the chairman appointed for each district shall have power to select associates, not exceeding four in number, to assist him in his labors.

*Resolved*, That the several state medical societies be requested to use their influence to procure the appointment by the legislatures of sanitory commissions.

(Signed) W. L. SUTTON,
JNO. L. ATLEE.
W. HOOKER,
JOSEPH PARRISH,
Z. PITCHER,
RO. W. HAXALL.

Dr. Storer, of Mass., sent to the secretary's table a correspondence between the president of the Epidemiological society of London and the Hon. Abbott Lawrence, ambassador to England, together with some documents relating to the organization and usefulness of the society.

On motion of Dr. Condie, of Pa., they were laid on the table.

A letter was received and read from Dr. Robley Dunglison, foreign secretary, and one of the vice presidents of the Sydenham society of London, presenting copies of the constitution and laws of the society; which, on Dr. Hayward's motion, were laid on the table.

Dr. Pope, of Mo., then read a report from the committee on the uses of water in surgery; which, on motion of Dr. Drake, was referred to the committee on publication.

On motion, the association then adjourned.

---

Friday, May 7, 1852.

The association was called to order by the president at 4½ o'clock P. M.

The minutes of yesterday's session were read, amended and approved.

On motion of Dr. Stille of Pennsylvania, the paper read by Dr. Drake of Kentucky, on the "Influence of Climatic Changes on Consumption," was referred to the committee of publication.

Dr. Atlee of Pennsylvania, offered the following preamble and resolution, which were unanimously adopted:

Whereas, it is the duty of patriotism to do homage to those who have been benefactors to their country; and whereas the medical profession in the United States, heretofore not wanting in patriotic feeling or action, desire to co-operate with the other public bodies and institutions of the country in rendering their profound reverence to the memory of him who was "first in peace, first in war, and first in the hearts of his countrymen;"

*Be it therefore resolved*, That a committee of five be appointed, whose duty it shall be to solicit subscriptions from members of the American Medical Association, for the purpose of procuring a suitable stone with an appropriate inscription, for insertion, in the name of this association, into the national monument to the memory of Washington, now in progress of erection at Washington city.

The chair announced the committee, to consist of Drs. Jos. L. Atlee, W. P. Johnston, Ro. W. Haxall, Alfred Stille and Gouverneur Emerson.

Dr. C. C. Cox, of Maryland, offered the following resolution, which was lost:

*Resolved*, That the committee on publication be and are hereby directed to distribute copies of the Transactions of this association, when printed, to the several booksellers in the principal cities of the Union, for the more convenient access of members entitled to the same, and also for the purpose of disposing of such copies as may remain on hand after the members shall have been supplied.

Dr. Corbin, of Virginia, called up his resolution offered on Wednesday, in regard to accrediting one member from each state represented in the association, to travel in Europe and to report upon foreign medical affairs to the association.

The resolution was adopted.

Dr. Phelps, of New York, then called up his amendment to the constitution proposed last year, to insert in article vii, p. 60, after the word "endeavors" the words "in reliance of divine guidance and support."

The motion so to amend was lost.

Drs. Flint, of Kentucky, and Hooker, of Connecticut, made motions in regard to the constitution, but they were withdrawn.

Dr. J. M. Smith, of New York, chairman of the committee on nominations, presented the following report, which, on motion of Dr. Corbin, of Virginia, was adopted:

The committee of nominations, in fulfilling the duty of their appointment, propose to continue most of the special committees appointed by the association in May, 1851, and to appoint several new special committees; they therefore submit the following list of chairmen of special committees, with the subjects to them committed: * * * *

[The names of persons composing the committees are deferred until the next No.]

The secretary then read a letter from Dr. S. D. Gross, of Kentucky, chairman of the committee "on the results of surgical operations for the relief of malignant diseases," regretting his inability, after strenuous exertions, to present a satisfactory report to the present meeting, and asking to be continued at the head of the same committee.

Dr. Paul Lajus, of Pa., offered the following resolution, which, after some debate, was lost:

*Resolved*, That a prize of $250 be awarded hereafter to the best prize essay, and that honorable mention be awarded to the four next best essays, provided they be worthy of that honor.

Dr. Wood, of Pa., then moved that instead of awarding five prizes of $50 each, annually, that the association hereafter grant two prizes of $100 each, for the two best essays. Carried.

On motion of Dr. Stille, of Pa., the report on proposed amendments to the constitution was recommitted to the committee, with instructions "so to amend it as to admit a representation of the army and navy, and to make other alterations. * * * * * *

The reports of the committees on scientific subjects being called for, Dr. Horner, of Pa., moved that they be read by their titles and referred to the committee of publication; which motion was adopted.

The following reports were then presented, read by their titles, and referred to the committee of publication:

"On the Toxicological and Medicinal properties of our Cryptogamic Plants," by F. Peyre Porcher, of S. C.

"On the Epidemics of New Jersey, Pennsylvania, Delaware and Maryland," by J. L. Atlee, of Pa.

"On the Epidemics of South Carolina, Georgia, Florida and Alabama," by Dr. W. M. Boling, of Ala.

Together with this report, which was handed in by Dr. Drake, of Ky., there was also presented a paper by Dr. D. J. Cain, of S. C.; which was ordered to be appended to the report when published.

"On the Epidemics of Mississippi, Louisiana, Texas and Arkansas," by Dr. Ed. H. Barton, of La.

"On the Epidemics of Ohio, Indiana and Michigan," by Dr. Geo. Mendenhall, of Ohio.

Dr. Stewart, of N. Y., then presented the report of the committee on the amendments to the constitution, and read the following additions which the committee had made since its recommitment:

To section 1, article 2, add "Delegates may also be received from the United States army and navy."

In section 6, article 2, add the words "Comprise six professors and" after "provided said faculty shall."

In section 6, add to 3d requisition on faculties, the words "and also where students are required to continue their attendance on the lectures until the close of the session."

Add section 7: "The medical faculty of the University of Virginia shall be entitled to representation in the association, notwithstanding that it is not composed of six professors, and that it does not require three years of study for its pupils, but only so long as the present peculiar system of instruction and examination practiced by that institution shall continue in force."

Add section 9: "Delegates representing the medical staff of the United States army or navy shall be appointed by the chiefs of the army and navy medical bureaux. The number of delegates so appointed shall be four from the army medical officers and an equal number from the navy medical officers."

After some discussion, and the failure of several motions to alter, lay on the table, etc., the report from the committee was accepted as amended by an unanimous vote, and the propositions (as seen on page 303 of third day's proceedings) were recommended to the next association as amendments to the constitution.

Dr. Bolton, of Va., then gave notice of the following amendment, which he should call up at the next meeting: Add to section 6, article 2, "Provided that such college require of its matriculates an adequate preliminary examination."

Dr. F. C. Stewart, of N. Y., moved the following preamble and resolutions, which were seconded by Dr. Pope, of Mo., and unanimously adopted:

Whereas the building in which this association has held its present session was gratuitously furnished by the proprietors: Therefore,

*Resolved*, That the cordial thanks of the "American Medical Association" be and the same are hereby tendered to the pastor and trustees of the United Presbyterian congregation of the city of Richmond, for the kindness and hospitality manifested by them in tendering to the association the free use of their church and lecture room.

*Resolved*, That a copy of these resolutions be signed by the president and secretaries of the association, and transmitted to the pastor and trustees of the United Presbyterian congregation.

Dr. Dunbar, of Md., offered the following resolution, which was unanimously adopted by a rising vote:

*Resolved*, That the thanks of this association are hereby voted to the president for the able and satisfactory manner in which he has presided over its meetings, and also to the secretaries for the faithful manner in which they have discharged their laborious duties.

On motion of Dr. Thompson, of Delaware, and seconded by Dr. Rogers, of Virginia, the following resolution was unanimously adopted, and a copy of it was directed to be transmitted to Dr. Moultrie:

*Resolved*, That the thanks of the association are unanimously voted to Dr. James Moultrie, of South Carolina, its late president, for the able, impartial and faithful manner in which he has discharged the duties of president of this association during the past year.

On motion of Dr. Gooch, of Va., the president was empowered to make the appointments under Dr. Corbin's resolution offered on the second day and passed, at any time during the year.

On motion of Dr. Pope, of Missouri, the association then adjourned to meet in May next, in the city of New York.

The vice president in the chair, Dr. T. Y. Simons, of S. C., then, in a few appropriate remarks congratulated the members on the happy termination of their meeting, and declared it adjourned *sine die.*

---

*Diluted Pyroligneous Acid as a Gargle.* By John Evans, M. D.

I have for several years been using diluted pyroligneous acid as a gargle in cases of inflammation of the fauces and tonsils with better success than any other article that I have prescribed.

I put a teaspoonful of the acid obtained from the shops into a wine glass of water, and direct the patient to gargle the throat frequently with it.

In the sore throat caused by exposure, so common throughout the country, it generally relieves the soreness and stiffness felt in swallowing very promptly.

In chronic inflammation, with or without ulceration of the throat, I have found it a very valuable remedy.

In the sore throat of scarlatina, it has generally afforded a very prompt amelioration of this symptom of the disease.

In several cases of habitual tonsilitis, by using this gargle freely at the commencement of the disease, I have been able to arrest the progress of the inflammation and secure a resolution.

Its use is not unpleasant; it is safe, even if used for hours continuously, and has an additional advantage in removing the fœtor of the breath.—*North Western Med. and Surg. Journal.*

